# What Role Should Government Play in the Personal Carbon Trading Market: Motivator or Punisher?

**DOI:** 10.3390/ijerph16111905

**Published:** 2019-05-29

**Authors:** Daoyan Guo, Hong Chen, Ruyin Long

**Affiliations:** 1 School of Management, China University of Mining and Technology, Xuzhou 221116, China; guodaoyan@163.com; 2 School of Management, Xi’an University of Science and Technology, Xi’an 710054, China

**Keywords:** personal carbon trading, downstream carbon emissions, government policy, evolutionary game, numerical simulation

## Abstract

With increasing downstream carbon emissions, the implementation of a personal carbon trading scheme is urgently required. In order to facilitate the progress, government departments are supposed to adopt a motivating or punitive policy to make guidance for downstream carbon emissions reduction. This study determined and verified the evolutionarily stable strategies (ESSs) of government departments and individuals whose carbon emissions exceeded the initial carbon allowance (CEEICA individuals) by using the evolutionary game and numerical simulation methods, respectively. The findings show that the ESS of government departments is always a punitive policy during the variation of strategies of CEEICA individuals. The ESS of CEEICA individuals is an active plan when the added cost (the difference between emissions reduction cost and trading earning) is less than the carbon tax; otherwise, it is a passive plan. Furthermore, the rate of convergence can be significantly influenced by the probabilistic distances between initial strategies and the ESSs. On the basis of these findings, this study suggested implementing a “punishment first, motivation-supplemented” policy, and developing a stable operational mechanism for a personal carbon trading market.

## 1. Introduction

On November 4, 2016, the Paris Agreement became legally effective as the first global agreement regarding the mitigation of climate change by nearly 200 countries and regions, where it indicated that low-carbon development, climatic adaptation, and sustainable development are the global consensus [1,2]. Numerous countries (e.g., European Union, Australia, Canada, Japan, and China) made commitments to reduce greenhouse gas emissions and to mitigate climate change in the Paris Agreement. In order to meet the targets in the Paris Agreement, many active measures have been taken by most countries. For example, some countries aim to develop renewable energy (e.g., solar energy and wind energy) and to generalize eco-friendly vehicles, such as hybrid vehicles, fuel cell vehicles, natural gas vehicles, and clean diesel vehicles [3,4]. It should be noted that the European Union, Australia, New Zealand, China, Korea, and some other countries have established carbon trading markets to reduce carbon emissions [5,6], and carbon trading schemes have become an important measure for reducing carbon emissions, but the existing carbon trading markets all focus on upstream sectors and corresponding government policies. The government departments of California implemented a Zero-Emission Vehicle (ZEV) Program to promote the use of zero-emission vehicles, where automobile manufacturers can earn credits by selling zero-emission cars and trucks, and the ZEV credits can be traded among manufacturers. The New South Wales (NSW) Greenhouse Gas Abatement Scheme imposed a strict performance guarantee where energy producers that exceeded their allotment of emissions could offset them by either surrendering the NSW Greenhouse Gas Abatement Certificate purchased from others in the scheme, or by paying an 11 AUD per tonne fine. Although the effects of “motivating policy” and “punitive policy” on the reduction of carbon emissions remain inconclusive, government departments obviously play crucial roles in the carbon trading market.

Due to improvements in living standards, the carbon emissions from household energy consumption are considered to be an important source of greenhouse gases. For example, more than 80% of the national carbon dioxide emissions in the United States were caused by consumer demands in 1997 [7]. In the United Kingdom, about 74% of the carbon dioxide emissions were related to household consumption in 2000 [8]. In China, household carbon emissions represented 35% of the national carbon emissions in 2007 [9]. In Singapore, household-related emissions accounted for about a quarter of the total carbon emissions from 2000 to 2010 [10]. Furthermore, the quantity and percentage of downstream carbon emissions exhibit increasing trends [11,12,13], which implies that individuals have a crucial role to play in reducing carbon emissions [7,14,15]. Carbon trading is an important measure for reducing carbon emissions [16,17,18,19], thus building a personal carbon trading market will be useful for allowing countries and regions throughout the world to fulfill their carbon emissions reduction targets. The reduction of downstream carbon emissions is calling for policy guidance, therefore, the question arises: what role should government departments play in the personal carbon trading market: motivator or punisher? The successful implementation of personal carbon trading schemes will inevitably involve a game between government departments and individuals whose carbon emissions exceeded the initial carbon allowance (CEEICA individuals).

The research of personal carbon trading mainly focuses on the characteristics of personal carbon trading, the carbon allowance schemes, and the implementation of personal carbon trading. The study of personal carbon trading originated in the middle 1990s when Fleming (1996) suggested that governments should set a carbon cap based on their carbon emissions reduction target as well as allocating some or all permits to individuals for household direct energy consumption and personal travel [20]. Thus, each adult can acquire an equal carbon emission allowance, but the quantity decreases each year [21,22]. Furthermore, there are several schemes for allocating initial carbon allowance: personal carbon allowance, cap and share, and tradable energy quotas [20,23,24,25]. There are some differences among these schemes (e.g., the sectors covered and proportions allocated), but they are all designed to provide guidance for individuals to improve their lifestyle and consumption pattern [26], and the carbon emissions reduction goal then can be achieved. The UK government firstly considered the personal carbon trading issue in 2003, where a proposal was made to introduce the personal carbon trading scheme. The scheme was not adopted because the Department for Environment, Food, and Rural Affairs found that the cost of personal carbon trading is so high that it cannot be accepted by residents at present, and thus it is a forward-looking scheme [27]. However, personal carbon trading schemes are characterized by efficiency and fairness [6,28,29]. The implementation of personal carbon trading schemes will increase the welfare of low-income residents, which is conducive to the promotion of social equity [30]. In addition, Guo, Chen, and Long (2019) showed that the heterogeneous emotions of the government and individuals could affect the equilibrium strategies regarding the pattern of implementing personal carbon trading schemes [6], which indicates that governments play an important role in the personal carbon trading market. In summary, the existing literature hasn’t found a policy solution for government departments to efficiently guide CEEICA individuals toward reducing their carbon emissions, and even the strategies that government departments and CEEICA individuals should follow to achieve a win-win result.

The purpose of this study is to determine and verify the evolutionarily stable strategies (ESSs) of government departments and CEEICA individuals for reducing downstream carbon emissions, further, provide valuable references for the implementation of personal carbon trading schemes. According to the analysis given above, government departments and CEEICA individuals were considered as bounded rational players in a personal carbon trading market, where government departments were programmed to follow a motivating or punitive policy to instruct CEEICA individuals to reduce carbon emissions, and CEEICA individuals were programmed to follow an active or passive plan to reduce carbon emissions. Next, replicator dynamics equations were established to analyze the evolutionary stability of the strategies adopted by government departments and CEEICA individuals, where the dynamic evolutionary process was simulated using MATLAB to verify the ESSs and to analyze the evolutionary rates of their strategies.

## 2. Establishment of the Evolutionary Game Model

### 2.1. Assumptions of the Evolutionary Game Model

The premise of personal carbon trading is that each person has equal permits of carbon emissions in the personal carbon trading market, which is derived from “contraction and convergence” and this embodies the equality of environmental policy [31]. The initial carbon allowance is equal for all individuals, and thus, CEEICA individuals must make careful decisions about reducing their carbon emissions or purchasing emission permits. Furthermore, government departments and CEEICA individuals are unable to adopt the optimal strategy to maximize their benefits due to the complex, diversified, and uncertain environment. Thus, government departments and CEEICA individuals are bounded rational players because they have to make a decision based on incomplete and asymmetric information.

An important responsibility for government departments in the personal carbon trading market is to decide the orientation of policy (i.e., “motivating policy” and “punitive policy”) to make guidance for downstream carbon emissions reduction. In the model, government departments are considered to have two optional strategies: a “motivating policy” ($G_{1}$) or “punitive policy” ($G_{2}$). When government departments adopt the “punitive policy”, CEEICA individuals need to pay a carbon tax if their actual carbon emissions are more than their initial carbon allowance. When government departments adopt the “motivating policy”, CEEICA individuals can get a subsidy if their actual carbon emissions are less than the initial carbon allowance. In addition, CEEICA individuals are considered to have two optional strategies: an “active plan” ($P_{1}$) or “passive plan” ($P_{2}$), where the “active plan” indicates CEEICA individuals purchasing low-carbon products or cutting back on high-carbon activities to make their actual carbon emissions lower than the initial carbon allowance, and the “passive plan” denotes CEEICA individuals living as usual or passively taking carbon emissions reduction measures so their actual carbon emissions still exceed the initial carbon allowance. The personal carbon trading market is considered to be a stable operation without trading losses. The initial carbon allowances of all individuals can be surrendered before the approval date or traded with other individuals, but they cannot be carried forward to the next period. The carbon price decided by the market is assumed to be stable in one period. The parameters in the game model are summarized in Table 1.

There are four combinations of strategies for government departments and CEEICA individuals: (motivating policy, active plan), (motivating policy, passive plan), (punitive policy active plan), and (punitive policy, passive plan). According to the parameters in Table 1, the payoff matrix for the evolutionary game is shown in Table 2.

### 2.2. Establishment of the Replicator Dynamic Equations

According to the evolutionary game theory, the replicator dynamic equation proposed by Taylor and Jonker (1978) represents the dynamic differential equation for the frequency of one strategy adopted by one population [32,33,34]. In the initial stage of the game, let x (0≤x≤1) be the probability of government departments following “motivating policy” strategy, 1−x is the probability of the following “punitive policy” strategy, y (0≤y≤1) is the probability of CEEICA individuals following the “active plan” strategy, and 1−y is the probability of the following “passive plan” strategy. Then, the replicator dynamic equations of the strategies adopted by government departments and CEEICA individuals can be established based on the above payoff matrix.

(1) Replicator dynamic equation for the strategies of government departments

The expected payoffs for the “motivating policy” strategy and “punitive policy” strategy are represented by $E_{11}$ and $E_{12}$, respectively, and the average expected payoff is represented by $E_{1}$. Hence,

(1) $$E_{11}=y\times \left[K_{1}\times w-\left(T_{A}-T_{R1}\right)\times s-C_{2}-\left(T_{A}-T_{R1}\right)\times p\right]\;+\left(1-y\right)\times \left[K_{2}\times w-C_{2}+\left(T_{R2}-T_{A}\right)\times p\right]$$

(2) $$E_{12}=y\times \left[K_{1}\times w-C_{2}-\left(T_{A}-T_{R1}\right)\times p\right]\;+\left(1-y\right)\times \left[K_{2}\times w-C_{2}+\left(T_{R2}-T_{A}\right)\times p+\left(T_{R2}-T_{A}\right)\times t\right]$$

(3) $$E_{1}=xE_{11}+\left(1-x\right)E_{12}.$$

The replicator dynamic equation for the strategies of government departments is established as follows:

(4) $$F\left(x\right)=\frac{dx}{dt}=x\left(E_{11}-E_{1}\right)=x\left(1-x\right)\times \left\{y\times \left[\left(T_{H}-T_{A}-K_{2}\right)\times t-\left(T_{A}+K_{1}-T_{H}\right)\times s\right]\;-\left(T_{H}-T_{A}-K_{2}\right)\times t\right\}.$$

(2) Replicator dynamic equation for strategies of CEEICA individuals

Let $E_{21}$, $E_{22}$, and $E_{2}$ be the expected payoffs for the “active plan” strategy, and the “passive plan” strategy, and the average expected payoff, respectively. Thus,

(5) $$E_{21}=x\times \left[\left(T_{A}-T_{R1}\right)\times s-K_{1}\times c-C_{1}+\left(T_{A}-T_{R1}\right)\times p\right]\;+\left(1-x\right)\times \left[-K_{1}\times c-C_{1}+\left(T_{A}-T_{R1}\right)\times p\right]$$

(6) $$E_{22}=x\times \left[-K_{2}\times c-C_{1}-\left(T_{R2}-T_{A}\right)\times p\right]\;+\left(1-x\right)\times \left[-K_{2}\times c-C_{1}-\left(T_{R2}-T_{A}\right)\times p-\left(T_{R2}-T_{A}\right)\times t\right]$$

(7) $$E_{2}=yE_{21}+\left(1-y\right)E_{22}.$$

The replicator dynamic equation for the strategies of CEEICA individuals is established as follows:

(8) $$F\left(y\right)=\frac{dy}{dt}=y\left(E_{21}-E_{2}\right)=y\left(1-y\right)\times \left\{x\times \left[\left(T_{A}+K_{1}-T_{H}\right)\times s-\left(T_{H}-T_{A}-K_{2}\right)\times t\right]\;+\left[-\left(K_{1}-K_{2}\right)\times c+\left(K_{1}-K_{2}\right)\times p+\left(T_{H}-T_{A}-K_{2}\right)\times t\right]\right\}.$$

## 3. Stability Analysis of the Evolutionary Game Model

The bounded rationality hypothesis assumes that government departments and CEEICA individuals cannot initially adopt the optimal strategy, so an asymptotic adjustment process is needed to achieve stability. Government departments and CEEICA individuals adjust their strategies continually according to their vested interests in order to improve their benefits. The ESS can only occur when the evolutionary system achieves equilibrium. Thus, this study will analyze the stability of the strategies adopted by government departments and CEEICA individuals.

### 3.1. Stability Analysis for the Strategy of Government Departments

Based on the analysis above (in Section 2.2), the replicator dynamic equation for the strategies of government departments is:

(9) $$F\left(x\right)=x\left(1-x\right)\times \left\{y\times \left[\left(T_{H}-T_{A}-K_{2}\right)\times t-\left(T_{A}+K_{1}-T_{H}\right)\times s\right]-\left(T_{H}-T_{A}-K_{2}\right)\times t\right\}.$$

(1) If $y=\frac{\left(T_{H}-T_{A}-K_{2}\right)\times t}{\left(T_{H}-T_{A}-K_{2}\right)\times t-\left(T_{A}+K_{1}-T_{H}\right)\times s}$, then F(x)=0. The evolutionary system will be stable for each x, i.e., both the “motivating policy” and “punitive policy” are the ESSs of government departments.

(2) If $y\neq \frac{\left(T_{H}-T_{A}-K_{2}\right)\times t}{\left(T_{H}-T_{A}-K_{2}\right)\times t-\left(T_{A}+K_{1}-T_{H}\right)\times s}$, and let F(x)=0, then x=0 or x=1. According to the stability theory for differential equations [35], x will be the ESS only when $\frac{dF\left(x\right)}{dx}<0$. By taking the derivative of F(x), then $\frac{dF\left(x\right)}{dx}=\left(1-2x\right)\times \left\{y\times \left[\left(T_{H}-T_{A}-K_{2}\right)\times t-\left(T_{A}+K_{1}-T_{H}\right)\times s\right]-\left(T_{H}-T_{A}-K_{2}\right)\times t\right\}$. According to the known parameter settings, there exists $T_{H}-T_{A}-K_{2}>0$, then $\left(T_{H}-T_{A}-K_{2}\right)\times t-\left(T_{A}+K_{1}-T_{H}\right)\times s$ needs to be further considered in different conditions.

If $\left(T_{H}-T_{A}-K_{2}\right)\times t-\left(T_{A}+K_{1}-T_{H}\right)\times s<0$, then $\frac{\left(T_{H}-T_{A}-K_{2}\right)\times t}{\left(T_{H}-T_{A}-K_{2}\right)\times t-\left(T_{A}+K_{1}-T_{H}\right)\times s}<0$, so it always has $y>\frac{\left(T_{H}-T_{A}-K_{2}\right)\times t}{\left(T_{H}-T_{A}-K_{2}\right)\times t-\left(T_{A}+K_{1}-T_{H}\right)\times s}$, and thus, x=0 is the ESS. Therefore, government departments with bounded rationality will play the “punitive plan” strategy when the government subsidy for CEEICA individuals is more than the carbon tax for CEEICA individuals.

If $\left(T_{H}-T_{A}-K_{2}\right)\times t-\left(T_{A}+K_{1}-T_{H}\right)\times s>0$, then $\frac{\left(T_{H}-T_{A}-K_{2}\right)\times t}{\left(T_{H}-T_{A}-K_{2}\right)\times t-\left(T_{A}+K_{1}-T_{H}\right)\times s}>1$, so it always has $y<\frac{\left(T_{H}-T_{A}-K_{2}\right)\times t}{\left(T_{H}-T_{A}-K_{2}\right)\times t-\left(T_{A}+K_{1}-T_{H}\right)\times s}$, and thus, x=0 is the ESS. Therefore, bounded rational government departments will play the “punitive plan” strategy when the government subsidy for CEEICA individuals is less than the carbon tax for CEEICA individuals.

### 3.2. Stability Analysis of the Strategy of CEEICA Individuals

Based on the analysis above (in Section 2.2), the replicator dynamic equation for the strategies of CEEICA individuals is: $F\left(y\right)=y\left(1-y\right)\times \left\{x\times \left[\left(T_{A}+K_{1}-T_{H}\right)\times s-\left(T_{H}-T_{A}-K_{2}\right)\times t\right]+\left[-\left(K_{1}-K_{2}\right)\times c+\left(K_{1}-K_{2}\right)\times p+\left(T_{H}-T_{A}-K_{2}\right)\times t\right]\right\}$.

(1) If $x=\frac{\left(K_{1}-K_{2}\right)\times c-\left(K_{1}-K_{2}\right)\times p-\left(T_{H}-T_{A}-K_{2}\right)\times t}{\left(T_{A}+K_{1}-T_{H}\right)\times s-\left(T_{H}-T_{A}-K_{2}\right)\times t}$, then F(y)=0. The evolutionary system will be stable for each y, i.e., both the “active plan” and “passive plan” are the ESSs of CEEICA individuals.

(2) If $x\neq \frac{\left(K_{1}-K_{2}\right)\times c-\left(K_{1}-K_{2}\right)\times p-\left(T_{H}-T_{A}-K_{2}\right)\times t}{\left(T_{A}+K_{1}-T_{H}\right)\times s-\left(T_{H}-T_{A}-K_{2}\right)\times t}$, and let F(y)=0, then y=0 or y=1. According to the stability theory for differential equations [35], y will be the ESS only when $\frac{dF\left(y\right)}{dy}<0$. By taking a derivative of F(y), it has $\frac{dF\left(y\right)}{dy}=\left(1-2y\right)\times \left\{x\times \left[\left(T_{A}+K_{1}-T_{H}\right)\times s-\left(T_{H}-T_{A}-K_{2}\right)\times t\right]+\left[-\left(K_{1}-K_{2}\right)\times c+\left(K_{1}-K_{2}\right)\times p+\left(T_{H}-T_{A}-K_{2}\right)\times t\right]\right\}$. As c−p>0 based on the above parameters settings, then $\frac{\left(K_{1}-K_{2}\right)\times c-\left(K_{1}-K_{2}\right)\times p-\left(T_{H}-T_{A}-K_{2}\right)\times t}{\left(T_{A}+K_{1}-T_{H}\right)\times s-\left(T_{H}-T_{A}-K_{2}\right)\times t}$ needs to be considered in different conditions.

(1) If $\frac{\left(K_{1}-K_{2}\right)\times c-\left(K_{1}-K_{2}\right)\times p-\left(T_{H}-T_{A}-K_{2}\right)\times t}{\left(T_{A}+K_{1}-T_{H}\right)\times s-\left(T_{H}-T_{A}-K_{2}\right)\times t}<0$, and it always has $x>\frac{\left(K_{1}-K_{2}\right)\times c-\left(K_{1}-K_{2}\right)\times p-\left(T_{H}-T_{A}-K_{2}\right)\times t}{\left(T_{A}+K_{1}-T_{H}\right)\times s-\left(T_{H}-T_{A}-K_{2}\right)\times t}$, then two conditions must be analyzed.

If $\left(K_{1}-K_{2}\right)\times c-\left(K_{1}-K_{2}\right)\times p>\left(T_{H}-T_{A}-K_{2}\right)\times t>\left(T_{A}+K_{1}-T_{H}\right)\times s$, then ${\frac{dF\left(y\right)}{dy}|}_{y=0}<0$, ${\frac{dF\left(y\right)}{dy}|}_{y=1}>0$, and thus, y=0 is the ESS. Therefore, CEEICA individuals with bounded rationality will play the “passive plan” strategy when the added cost (the difference between the emissions reduction cost and trading earning) is more than the carbon tax and the carbon tax is simultaneously more than the government subsidy for CEEICA individuals.

If $\left(K_{1}-K_{2}\right)\times c-\left(K_{1}-K_{2}\right)\times p<\left(T_{H}-T_{A}-K_{2}\right)\times t<\left(T_{A}+K_{1}-T_{H}\right)\times s$, then ${\frac{dF\left(y\right)}{dy}|}_{y=0}>0$, ${\frac{dF\left(y\right)}{dy}|}_{y=1}<0$, and thus, y=1 is the ESS. Therefore, bounded rational CEEICA individuals will play the “active plan” strategy when the added cost is less than the carbon tax and the carbon tax is simultaneously less than the government subsidy.

(2) If $\frac{\left(K_{1}-K_{2}\right)\times c-\left(K_{1}-K_{2}\right)\times p-\left(T_{H}-T_{A}-K_{2}\right)\times t}{\left(T_{A}+K_{1}-T_{H}\right)\times s-\left(T_{H}-T_{A}-K_{2}\right)\times t}>0$, then two conditions should be further analyzed.

If $x>\frac{\left(K_{1}-K_{2}\right)\times c-\left(K_{1}-K_{2}\right)\times p-\left(T_{H}-T_{A}-K_{2}\right)\times t}{\left(T_{A}+K_{1}-T_{H}\right)\times s-\left(T_{H}-T_{A}-K_{2}\right)\times t}$, then ${\frac{dF\left(y\right)}{dy}|}_{y=0}>0$, ${\frac{dF\left(y\right)}{dy}|}_{y=1}<0$, and thus, y=1 is the ESS. CEEICA individuals will play the “active plan” strategy in this condition.

If $x<\frac{\left(K_{1}-K_{2}\right)\times c-\left(K_{1}-K_{2}\right)\times p-\left(T_{H}-T_{A}-K_{2}\right)\times t}{\left(T_{A}+K_{1}-T_{H}\right)\times s-\left(T_{H}-T_{A}-K_{2}\right)\times t}$, then ${\frac{dF\left(y\right)}{dy}|}_{y=0}<0$, ${\frac{dF\left(y\right)}{dy}|}_{y=1}>0$, and thus, y=0 is the ESS. CEEICA individuals will play the “passive plan” strategy in this condition.

### 3.3. Stability Analysis for Their Strategies

According to the analysis in Section 3.1 and Section 3.2, there are five equilibrium points comprising: $B_{1}\left(0,0\right)$, $B_{2}\left(1,0\right)$, $B_{3}\left(0,1\right)$, $B_{4}\left(1,1\right)$, and $B_{5}\left(X_{0},Y_{0}\right)$, and $X_{0}=\frac{\left(K_{1}-K_{2}\right)\times c-\left(K_{1}-K_{2}\right)\times p-\left(T_{H}-T_{A}-K_{2}\right)\times t}{\left(T_{A}+K_{1}-T_{H}\right)\times s-\left(T_{H}-T_{A}-K_{2}\right)\times t}$, $Y_{0}=\frac{\left(T_{H}-T_{A}-K_{2}\right)\times t}{\left(T_{H}-T_{A}-K_{2}\right)\times t-\left(T_{A}+K_{1}-T_{H}\right)\times s}$. The stability of these equilibrium points can be analyzed based on the local stability of Jacobian matrix [36]. The determinant of the Jacobian matrix is expressed as $Det\left(J\right)=\frac{\partial F\left(x\right)}{\partial x}\times \frac{\partial F\left(y\right)}{\partial y}-\frac{\partial F\left(x\right)}{\partial y}\times \frac{\partial F\left(y\right)}{\partial x}$, and the trace of the Jacobian matrix is expressed as $Tr\left(J\right)=\frac{\partial F\left(x\right)}{\partial x}+\frac{\partial F\left(y\right)}{\partial y}$. The equilibrium point can only be the local asymptotically stable point in a discrete system in the case when Det(J)>0 and Tr(J)<0, where the corresponding strategy is the ESS. Thus, after substituting the equilibrium points given above into the expressions for Det(J) and Tr(J), the results are shown in Table 3. It is shown that there are two ESSs under the six conditions based on the results of the evolutionary stability analysis.

(1) If $\left(K_{1}-K_{2}\right)\times c-\left(K_{1}-K_{2}\right)\times p<\left(T_{H}-T_{A}-K_{2}\right)\times t<\left(T_{A}+K_{1}-T_{H}\right)\times s$, or $\left(K_{1}-K_{2}\right)\times c-\left(K_{1}-K_{2}\right)\times p<\left(T_{A}+K_{1}-T_{H}\right)\times s<\left(T_{H}-T_{A}-K_{2}\right)\times t$, or $\left(T_{H}-T_{A}-K_{2}\right)\times t>\left(K_{1}-K_{2}\right)\times c-\left(K_{1}-K_{2}\right)\times p>\left(T_{A}+K_{1}-T_{H}\right)\times s$, then $B_{3}\left(0,1\right)$ will be the ESSs, i.e., government departments will adopt a punitive policy and CEEICA individuals will adopt an active plan after a long period of evolution.

(2) If $\left(K_{1}-K_{2}\right)\times c-\left(K_{1}-K_{2}\right)\times p>\left(T_{A}+K_{1}-T_{H}\right)\times s>\left(T_{H}-T_{A}-K_{2}\right)\times t$, or $\left(T_{H}-T_{A}-K_{2}\right)\times t<\left(K_{1}-K_{2}\right)\times c-\left(K_{1}-K_{2}\right)\times p<\left(T_{A}+K_{1}-T_{H}\right)\times s$, or $\left(K_{1}-K_{2}\right)\times c-\left(K_{1}-K_{2}\right)\times p>\left(T_{H}-T_{A}-K_{2}\right)\times t>\left(T_{A}+K_{1}-T_{H}\right)\times s$, then $B_{1}\left(0,0\right)$ will be the ESS. Therefore, government departments will adopt a punitive policy and CEEICA individuals will adopt a passive plan after a long period of evolution.

## 4. Numerical Simulation

To visually verify the evolutionary stability of their strategies under different conditions, MATLAB R2012a was used to simulate the dynamic evolutionary process and to analyze the effects of variations in the parameters on the evolutionary results. Thus, each parameter was assigned a value as follows: $T_{A}=6$, $T_{H}=10$, $K_{1}=4.5$, $K_{2}=2.5$, and c=0.30.

**Figure 1:**
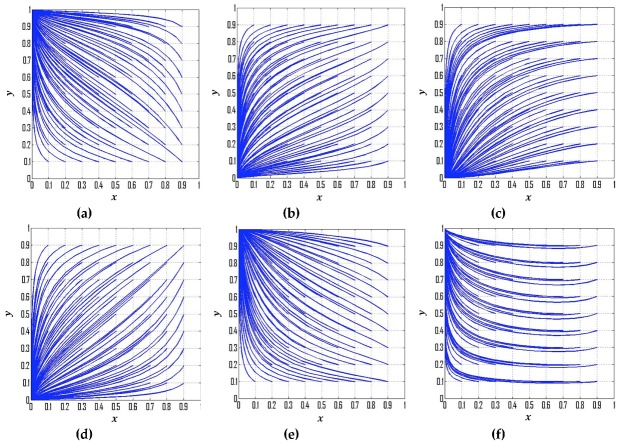
Dynamic evolutionary paths for the strategies of government departments and CEEICA individuals: (**a**) Condition No. 1; (**b**) Condition No. 2; (**c**) Condition No. 3; (**d**) Condition No. 4; (**e**) Condition No. 5; and (**f**) Condition No. 6.

**Figure 2:**
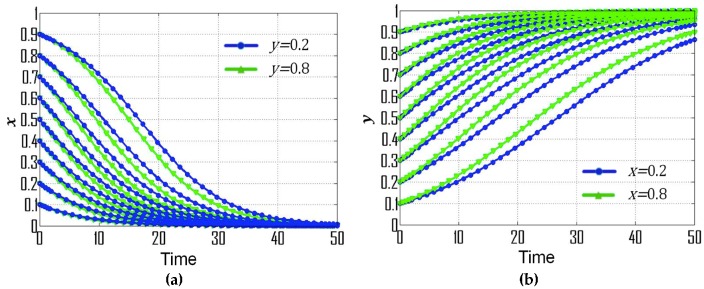
Dynamic evolutionary paths for each of the strategies under condition No. 1: (**a**) Strategies of government departments; (**b**) Strategies of CEEICA individuals.

**Figure 3:**
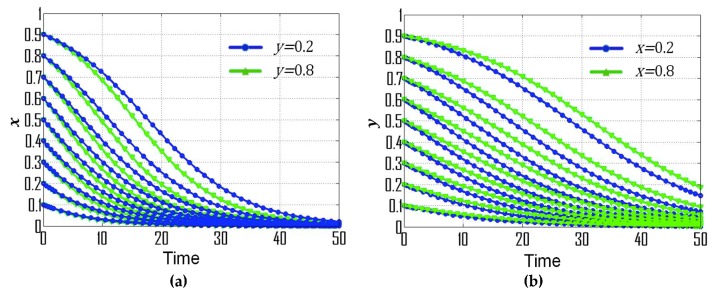
Dynamic evolutionary paths for each of the strategies under condition No. 2: (**a**) Strategies of government departments; (**b**) Strategies of CEEICA individuals.

**Figure 4:**
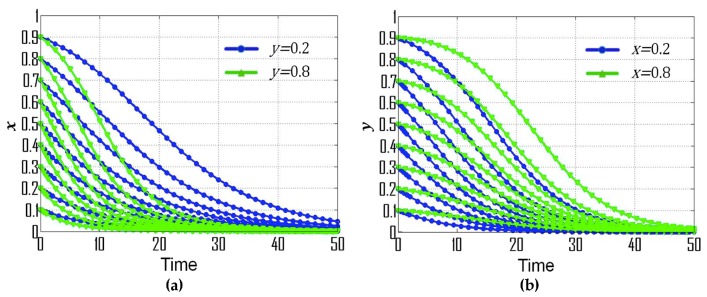
Dynamic evolutionary paths for each of the strategies under condition No. 3: (**a**) Strategies of government departments; (**b**) Strategies of CEEICA individuals.

**Figure 5:**
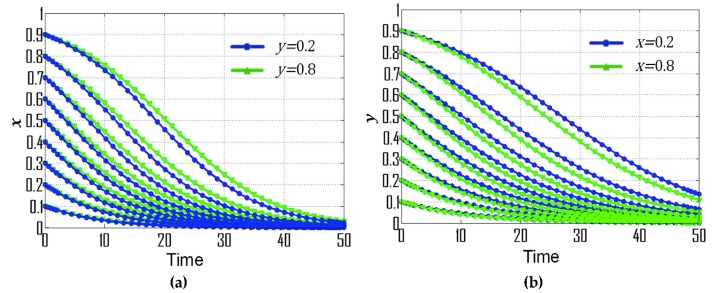
Dynamic evolutionary paths for each of the strategies under condition No. 4: (**a**) Strategies of government departments; (**b**) Strategies of CEEICA individuals.

**Figure 6:**
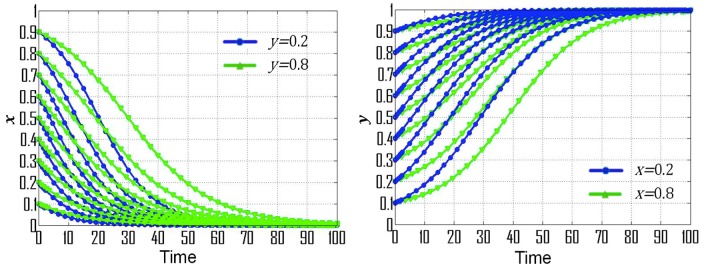
Dynamic evolutionary paths for each of the strategies under condition No. 5: (**a**) Strategies of government departments; (**b**) Strategies of CEEICA individuals.

**Figure 7:**
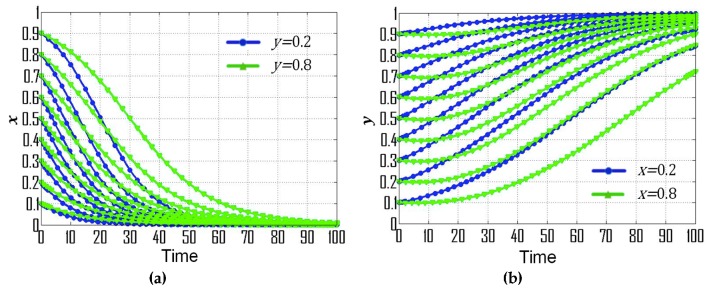
Dynamic evolutionary paths for each of the strategies under condition No. 6: (**a**) Strategies of government departments; (**b**) Strategies of CEEICA individuals.

(1) If p=0.28, s=0.30, and t=0.08, then the replicator dynamic equations for this special case are: F(x)=x(1−x)(−0.03y−0.12), F(y)=y(1−y)(0.03x+0.08), $\left(X_{0},Y_{0}\right)=\left(-\frac{8}{3},-4\right)$, which is consistent with condition No. 1. According to the dynamic evolutionary paths of their strategies (Figure 1a), point (0,1) is the ESS, which indicates that the ESS of government departments and CEEICA individuals are the punitive policy and the active plan, respectively. Figure 2 exemplifies the dynamic evolutionary paths for each of the strategies. In Figure 2a, a series of 0.8 (green line) and 0.2 (blue line) curves are paired according to the x value (the probability of playing the “motivating policy” strategy). In each pair, the point values of the 0.8 curves are constantly smaller than those of the 0.2 curves, indicating the rate of convergence of the government departments’ strategies will increase when there is a shorter probabilistic distance between the initial strategy of CEEICA individuals and an active plan. It can also be seen that a declining x value can shorten the convergence time of curves, which implies the rate of convergence is increasing with a dropping probability of the following “motivating policy” strategy. In Figure 2b, a series of 0.8 (green line) and 0.2 (blue line) curves are paired based on a y value (the probability of playing the “active policy” strategy). In each pair, the point values of the 0.8 curves are constantly larger than those of the 0.2 curves, indicating the rate of convergence of CEEICA individuals’ strategies will increase when there is a shorter probabilistic distance between the initial strategy of the government department and a motivating policy. It should be noted that rising y values can shorten the convergence time of curves, which implies the rate of convergence is increasing with a raising probability of the following “active plan” strategy.

(2) If p=0.20, *s* = 0.30, t=0.08, then F(x)=x(1−x)(−0.03y−0.12), F(y)=y(1−y)(0.03x−0.08), $\left(X_{0},Y_{0}\right)=\left(\frac{8}{3},-4\right)$, which is consistent with condition No. 2. Figure 1b exemplifies the dynamic evolutionary path for the strategies of government departments and CEEICA individuals under condition No. 2, where point (0,0) is the ESS, thus the ESS of government departments and CEEICA individuals are the punitive policy and the passive plan, respectively. Dynamic evolutionary paths for each of the strategies under condition No. 2 are shown in Figure 3. According to Figure 3a, the rate of convergence of government departments’ strategies increases when there is a shorter probabilistic distance between the initial strategy of CEEICA individuals and an active plan, and the rate of convergence also increases with a dropping probability of the following “motivating policy” strategy. In Figure 3b, it can be observed that the rate of convergence of CEEICA individuals’ strategies increases when there is a shorter probabilistic distance between the initial strategy of the government department and a punitive policy, and the rate of convergence also increases with a dropping probability of the following “passive plan” strategy.

(3) If p=0.18, s=0.50, t=0.06, then  F(x)=x(1−x)(−0.16y−0.09), F(y)=y(1−y)(0.16x−0.15), $\left(X_{0},Y_{0}\right)=\left(\frac{15}{16},-\frac{9}{16}\right)$, which is consistent with condition No. 3. It is obvious that point (0,0) is the ESS based on Figure 1c, which means the ESS of government departments and CEEICA individuals are the punitive policy and passive plan, respectively. Furthermore, Figure 4 exemplifies the dynamic evolutionary paths for each of the strategies under condition No. 3. In Figure 4a, it can be concluded that the rate of convergence of government departments’ strategies increases when there is a shorter probabilistic distance between the initial strategy of CEEICA individuals and an active plan, and it also increases with a dropping probability of the following “motivating policy” strategy. In addition, it is notable that the rate of convergence of CEEICA individuals’ strategies (Figure 4b) will decrease when there is a shorter probabilistic distance between the initial strategy of the government department and a motivating policy, and the rate of convergence also decreases with an increase in the probability of the following “passive plan” strategy.

(4) If p=0.20, s=0.20, t=0.08, then F(x)=x(1−x)(0.02y−0.12), F(y)=y(1−y)(−0.02x−0.08), $\left(X_{0},Y_{0}\right)=\left(-4,6\right)$, which is consistent with condition No. 4. According to the dynamic evolutionary paths for the strategies of government departments and CEEICA individuals under condition No. 4 shown in Figure 1d, the ESS of government departments and CEEICA individuals are proven to be the punitive policy and passive plan, respectively. Dynamic evolutionary paths for each of the strategies under condition No. 4 are exhibited in Figure 5. It can be concluded from Figure 5a that the rate of convergence of government departments’ strategies decreases when there is a shorter probabilistic distance between the initial strategy of CEEICA individuals and an active plan, and the rate of convergence also decreases with an increasing probability of the following “punitive policy” strategy. According to Figure 5b, the rate of convergence of CEEICA individuals’ strategies increases when there is a shorter probabilistic distance between the initial strategy of government department and a motivating policy, and the rate of convergence also increases with a dropping probability of the following “active plan” strategy.

(5) If p=0.28, s=0.12, t=0.08, then F(x)=x(1−x)(0.06y−0.12), F(y)=y(1−y)(−0.06x+0.08), $\left(X_{0},Y_{0}\right)=\left(\frac{4}{3},2\right)$, which is consistent with condition No. 5. It can be clearly seen that point (0,1) is the ESS based on the dynamic evolutionary paths of their strategies under condition No. 5 (Figure 1e), which implies that the respective ESS of the government departments and CEEICA individuals are the punitive policy and active plan, respectively. Furthermore, Figure 6 exemplifies the dynamic evolutionary paths for each of the strategies condition No. 5. According to Figure 6a, the rate of convergence of government departments’ strategies increases when there is a shorter probabilistic distance between the initial strategy of CEEICA individuals and a passive plan, and the rate of convergence increases with a dropping probability of the following “motivating policy” strategy. In Figure 6b, the rate of convergence of CEEICA individuals’ strategies increases when there is a shorter probabilistic distance between the initial strategy of government department and a punitive policy, and the rate of convergence increases with a raising probability of the following “active plan” strategy.

(6) If p=0.26, s=0.12, t=0.08, then F(x)=x(1−x)(0.06y−0.12), F(y)=y(1−y)(−0.06x+0.04), $\left(X_{0},Y_{0}\right)=\left(\frac{2}{3},2\right)$, which is consistent with condition No. 6. The dynamic evolutionary paths for their strategies under condition No. 6 are described in Figure 1f, which demonstrates that the ESS of government departments and CEEICA individuals are the punitive policy and active plan, respectively. Figure 7a exemplifies the dynamic evolutionary paths for the strategies of government departments under condition No. 6. Findings show that the rate of convergence of government departments’ strategies increases when there is a shorter probabilistic distance between the initial strategy of CEEICA individuals and a passive plan, and the rate of convergence increases with a dropping probability of the following “motivating policy” strategy. Figure 7b depicts the dynamic evolutionary paths for the strategies of CEEICA individuals. It can be found that the rate of convergence of CEEICA individuals’ strategies increases when there is a longer probabilistic distance between the initial strategy of government department and a motivating policy, and the rate of convergence decreases with a dropping probability of the following “active plan” strategy.

In summary, the rate of convergence can be affected by the probabilistic distance between the initial strategy of the government departments and the ESS, and the probabilistic distance between the initial strategy of CEEICA individuals and the ESS. With respect to the strategies of government departments, the rate of convergence will increase when there is a shorter probabilistic distance between the initial strategy of CEEICA individuals and the “active plan” strategy in the case where $\left(T_{A}+K_{1}-T_{H}\right)\times s>\left(T_{H}-T_{A}-K_{2}\right)\times t$, but it will decrease when there is a shorter probabilistic distance between the initial strategy of CEEICA individuals and the “active plan” strategy in the case where $\left(T_{A}+K_{1}-T_{H}\right)\times s<\left(T_{H}-T_{A}-K_{2}\right)\times t$. For the strategies of CEEICA individuals, the rate of convergence increases when there is a shorter probabilistic distance between the initial strategy of government departments and the “punitive policy” strategy in the case where $\frac{\left(K_{1}-K_{2}\right)\times c-\left(K_{1}-K_{2}\right)\times p-\left(T_{H}-T_{A}-K_{2}\right)\times t}{\left(T_{A}+K_{1}-T_{H}\right)\times s-\left(T_{H}-T_{A}-K_{2}\right)\times t}<0$, but it decreases when there is a shorter probabilistic distance between the initial strategy of government departments and the “punitive policy” strategy in the case where $\frac{\left(K_{1}-K_{2}\right)\times c-\left(K_{1}-K_{2}\right)\times p-\left(T_{H}-T_{A}-K_{2}\right)\times t}{\left(T_{A}+K_{1}-T_{H}\right)\times s-\left(T_{H}-T_{A}-K_{2}\right)\times t}>0$. In addition, a shorter probabilistic distance between the initial strategy and the ESS of government departments and CEEICA individuals accelerates the convergence of their strategies.

## 5. Discussion

The implementation of a personal carbon trading scheme can potentially contribute to reducing downstream carbon emissions, but the original intention is to effectively make guidance for individuals to live in a low-carbon lifestyle [37], thus CEEICA individuals are expected to adopt an active plan. The findings indicate that CEEICA individuals will definitely adopt a passive plan under condition Nos. 2, 3, and 4 (see Table 3), and thus these are unexpected results for government departments. In fact, CEEICA individuals can only play the “active plan” strategy under condition Nos. 1, 5, and 6. Furthermore, the relational expression of $\left(K_{1}-K_{2}\right)\times c-\left(K_{1}-K_{2}\right)\times p<\left(T_{H}-T_{A}-K_{2}\right)\times t$ is the same for these three conditions, i.e., CEEICA individuals with bounded rationality only follow the “active plan” strategy when the added cost (the difference between the emissions reduction cost and trading earning) is less than the carbon tax. Therefore, government departments can drive CEEICA individuals to reduce their carbon emissions by increasing the carbon tax because the emissions reduction cost c is generally fixed and the carbon price p, decided by the personal carbon trading market is steady during one period. In addition, the ESS of government departments is a punitive policy, which indicates that the carbon tax t per unit of carbon emissions are collected as the actual carbon emissions of CEEICA individuals is more than the initial carbon allowance. Coincidentally, some studies have suggested that a carbon tax can effectively reduce residents’ carbon emissions [15,38]. Preceding analyses show that a punitive policy is effective for downstream carbon emissions reduction, but the collection of the carbon tax will inevitably increase the financial burden of residents. Thus, it is very hard for government departments to make a decision on how to instruct CEEICA individuals to reduce carbon emissions in the personal carbon trading market. In any case, the results of this study provide theoretical support for government departments to adopt a punitive policy from the perspective of an evolutionary game model.

## 6. Conclusions and Policy Suggestions

### 6.1. Conclusions

This study determined and verified the ESSs of government departments and CEEICA individuals in the personal carbon trading market, and the main conclusions are outlined as follows: (1) The ESS of government departments is always a punitive policy under six conditions; (2) There are two ESSs of CEEICA individuals, one is an active plan when the added cost of the following “active plan” strategy is less than the carbon tax of the following “passive plan” strategy, and the other is a passive plan strategy when the added cost is more than the carbon tax; (3) When the subsidy is more than the carbon tax, a shorter probabilistic distance between the initial strategy of CEEICA individuals and an active plan induces an increase of the rate of convergence of the government departments’ strategies; as the subsidy is less than the carbon tax, a decrease can be observed; (4) When carbon tax is in the range between added cost and subsidy, the rate of convergence of CEEICA individuals’ strategies exhibits an increase with the shortening probabilistic distance between the initial strategy of government departments and a punitive policy; as the carbon tax is out of the range, a decrease is presented; and (5) A shorter probabilistic distance between the initial strategy and the ESS of government departments and CEEICA individuals accelerates the convergence of their strategies.

### 6.2. Policy Suggestions

To achieve a win-win result for government departments and CEEICA individuals in the personal carbon trading market as well as meeting the carbon emissions reduction target as early as possible, the following suggestions are proposed for government departments.

(1) Implement a “punishment first, motivation-supplemented” policy

A punitive policy should be theoretically implemented as it is the ESS of government departments. However, if the punitive policy is the only policy implemented by government departments, individuals may feel resentful and angry as it is a harsh policy that aims to reduce carbon emissions by collecting a heavy carbon tax from CEEICA individuals, which is not conducive to social stability. Therefore, a “punishment first, motivation-supplemented” policy should be implemented, and government departments can exert pressure on CEEICA individuals via a carbon tax as well as encouraging CEEICA individuals to actively reduce their carbon emissions by providing a government subsidy. In addition, government departments should consider the economic levels of different regions as well as household size and income in order to implement a reasonable “punishment first, motivation-supplemented” policy.

(2) Develop a stable operational mechanism for personal carbon trading market

**Figure 8:**
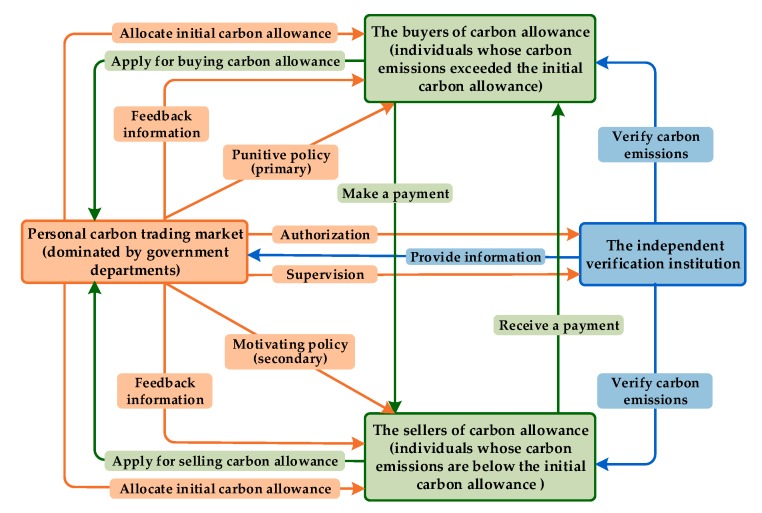
Operational process of the personal carbon trading market.

The above ESSs are determined based on the assumption of stable operation of the personal carbon trading market, thus, a stable operational mechanism is important and necessary for a personal carbon trading market. From the perspective of stability, the operational process of the personal carbon trading market is described as follows (Figure 8). First, government departments allocate an initial carbon allowance to each individual based on the cap-and-trade system and equity rule. Second, CEEICA individuals decide to adopt an active or passive plan by considering the policies of the government departments (e.g., punitive policy or motivating policy) and other factors (e.g., social atmosphere, climate and environment, and personal characteristics). Third, an independent verification institution verifies the actual carbon emissions of individuals and provides related information to government departments. Finally, CEEICA individuals apply to buy (or sell) carbon allowance, and they then trade with sellers (buyers). In addition, the verification process for the actual carbon emissions of individuals should be strictly supervised and carbon emissions information should be fed back rapidly to individuals, especially CEEICA individuals.

## Figures and Tables

**Table 1:** Summary of parameters in the game model.

Symbol	Meaning
$T_{A}$	Initial carbon allowance of CEEICA individuals
$T_{H}$	History carbon emissions of CEEICA individuals (i.e., the carbon dioxide emitted by CEEICA individuals in the last period) ($T_{H}>T_{A}$).
p	Carbon price in the personal carbon trading market
$K_{N}$	Reduced carbon emissions of CEEICA individuals ($K_{N}$ times per unit of carbon emission reduction, N=1, 2), where $K_{1}$ and $K_{2}$ represent the emission reductions obtained by following the “active plan” and “passive plan” strategy, respectively.
$T_{RN}$	Actual carbon emissions of CEEICA individuals ($T_{RN}=T_{H}-K_{N}$, N=1, 2), where $T_{R1}$ and $T_{R2}$ represent the emissions obtained by following the “active plan” and “passive plan” strategy, respectively ($T_{R2}>T_{A}>T_{R1}$).
$C_{1}$	Fixed cost for CEEICA individuals to participate in personal carbon trading (e.g., the cost for employing an independent party to validate or verify carbon emissions).
$C_{2}$	Fixed cost for government departments for policy making and market management
*c*	Cost of CEEICA individuals for reducing their per unit carbon emissions.
w	Public welfare obtained from the per unit carbon emissions reduction by CEEICA individuals (e.g., mitigating climate change and reducing energy consumption).
s	Subsidy for the per unit carbon emissions reduction when the actual carbon emissions of CEEICA individuals are less than the initial carbon allowance (i.e., the cost for government departments who played the “motivating policy”).
t	Carbon tax for the per unit carbon emissions when the actual carbon emissions of CEEICA individuals exceeded the initial carbon allowance (i.e., the benefit for government departments who played the “punitive policy”)

**Table 2:** Payoff matrix for the evolutionary game.

Strategy Combinations	Payoff of Government Departments	Payoff of CEEICA Individuals
(G_1_, P_1_)	$K_{1}\times w-\left(T_{A}-T_{R1}\right)\times s-C_{2}-\left(T_{A}-T_{R1}\right)\times p$	$\left(T_{A}-T_{R1}\right)\times s-K_{1}\times c-C_{1}+\left(T_{A}-T_{R1}\right)\times p$
(G_1_, P_2_)	$K_{2}\times w-C_{2}+\left(T_{R2}-T_{A}\right)\times p$	$-K_{2}\times c-C_{1}-\left(T_{R2}-T_{A}\right)\times p$
(G_2_, P_1_)	$K_{1}\times w-C_{2}-\left(T_{A}-T_{R1}\right)\times p$	$-K_{1}\times c-C_{1}+\left(T_{A}-T_{R1}\right)\times p$
(G_2_, P_2_)	$K_{2}\times w-C_{2}+\left(T_{R2}-T_{A}\right)\times p+\left(T_{R2}-T_{A}\right)\times t$	$-K_{2}\times c-C_{1}-\left(T_{R2}-T_{A}\right)\times p-\left(T_{R2}-T_{A}\right)\times t$

**Table 3:** Evolutionary stability analysis for their strategies.

No.	Condition	(*X*_0_,*Y*_0_)	Equilibrium Point	Det(J)	Tr(J)	Result
1	$\left(K_{1}-K_{2}\right)\times c-\left(K_{1}-K_{2}\right)\times p<\left(T_{H}-T_{A}-K_{2}\right)\times t<\left(T_{A}+K_{1}-T_{H}\right)\times s$	$X_{0}<0$ $Y_{0}<0$	$B_{1}\left(0,0\right)$	<0	Uncertain	SP
			$B_{2}\left(1,0\right)$	>0	>0	UP
			$B_{3}\left(0,1\right)$	>0	<0	ESS
			$B_{4}\left(1,1\right)$	<0	Uncertain	SP
2	$\left(K_{1}-K_{2}\right)\times c-\left(K_{1}-K_{2}\right)\times p>\left(T_{A}+K_{1}-T_{H}\right)\times s>\left(T_{H}-T_{A}-K_{2}\right)\times t$	$X_{0}>1$ $Y_{0}<0$	$B_{1}\left(0,0\right)$	>0	<0	ESS
			$B_{2}\left(1,0\right)$	<0	Uncertain	SP
			$B_{3}\left(0,1\right)$	<0	Uncertain	SP
			$B_{4}\left(1,1\right)$	>0	>0	UP
3	$\left(T_{H}-T_{A}-K_{2}\right)\times t<\left(K_{1}-K_{2}\right)\times c-\left(K_{1}-K_{2}\right)\times p<\left(T_{A}+K_{1}-T_{H}\right)\times s$	$0<X_{0}<1$ $Y_{0}<0$	$B_{1}\left(0,0\right)$	>0	<0	ESS
			$B_{2}\left(1,0\right)$	>0	>0	UP
			$B_{3}\left(0,1\right)$	<0	<0	SP
			$B_{4}\left(1,1\right)$	<0	>0	SP
4	$\left(K_{1}-K_{2}\right)\times c-\left(K_{1}-K_{2}\right)\times p>\left(T_{H}-T_{A}-K_{2}\right)\times t>\left(T_{A}+K_{1}-T_{H}\right)\times s$	$X_{0}<0$ $Y_{0}>1$	$B_{1}\left(0,0\right)$	>0	<0	ESS
			$B_{2}\left(1,0\right)$	<0	Uncertain	SP
			$B_{3}\left(0,1\right)$	<0	Uncertain	SP
			$B_{4}\left(1,1\right)$	>0	>0	UP
5	$\left(K_{1}-K_{2}\right)\times c-\left(K_{1}-K_{2}\right)\times p<\left(T_{A}+K_{1}-T_{H}\right)\times s<\left(T_{H}-T_{A}-K_{2}\right)\times t$	$X_{0}>1$ $Y_{0}>1$	$B_{1}\left(0,0\right)$	<0	Uncertain	SP
			$B_{2}\left(1,0\right)$	>0	>0	UP
			$B_{3}\left(0,1\right)$	>0	<0	ESS
			$B_{4}\left(1,1\right)$	<0	Uncertain	SP
6	$\left(T_{H}-T_{A}-K_{2}\right)\times t>\left(K_{1}-K_{2}\right)\times c-\left(K_{1}-K_{2}\right)\times p>\left(T_{A}+K_{1}-T_{H}\right)\times s$	$0<X_{0}<1$ $Y_{0}>1$	$B_{1}\left(0,0\right)$	<0	<0	SP
			$B_{2}\left(1,0\right)$	<0	>0	SP
			$B_{3}\left(0,1\right)$	>0	<0	ESS
			$B_{4}\left(1,1\right)$	>0	>0	UP

Note: SP indicates saddle point; UP indicates unstable point.

## References

[1] Lemieux C.J., Scott D.J. (2011). Changing climate, challenging choices: Identifying and evaluating climate change adaptation options for protected areas management in Ontario, Canada. Environ. Manag..

[2] Guo D., Chen H., Long R. (2018). Can China fulfill its commitment to reducing carbon dioxide emissions in the Paris Agreement? Analysis based on a back-propagation neural network. Environ. Sci. Pollut. Res..

[3] Jeong N.T., Yang S.M., Kim K.S., Wang M.S., Kim H.S., Suh M.W. (2016). Urban driving cycle for performance evaluation of electric vehicles. Int. J. Automot. Technol..

[4] Heidenreich S., Spieth P., Petschnig M. (2017). Ready, steady, green: Examining the effectiveness of external policies to enhance the adoption of eco-friendly innovations. J. Prod. Innov. Manag..

[5] Jin L., Duan K., Shi C., Ju X. (2017). The impact of technological progress in the energy sector on carbon emissions: An empirical analysis from China. Int. J. Environ. Res. Public Health.

[6] Guo D., Chen H., Long R. (2019). How to involve individuals in personal carbon trading? A game model taking into account the heterogeneous emotions of government and individuals. Nat. Hazards.

[7] Bin S., Dowlatabadi H. (2005). Consumer lifestyle approach to US energy use and the related CO_2_ emissions. Energy Policy.

[8] Baiocchi G., Minx J., Hubacek K. (2010). The impact of social factors and consumer behavior on carbon dioxide emissions in the United Kingdom. J. Ind. Ecol..

[9] Tian X., Chang M., Lin C., Tanikawa H. (2014). China’s carbon footprint: A regional perspective on the effect of transitions in consumption and production patterns. Appl. Energy.

[10] Su B., Ang B.W., Li Y. (2017). Input-output and structural decomposition analysis of Singapore’s carbon emissions. Energy Policy.

[11] Jones C., Kammen D.M. (2014). Spatial distribution of U.S. household carbon footprints reveals suburbanization undermines greenhouse gas benefits of urban population density. Environ. Sci. Technol..

[12] Ye H., Ren Q., Hu X., Lin T., Xu L., Li X., Zhang G., Shi L., Pan B. (2017). Low-carbon behavior approaches for reducing direct carbon emissions: Household energy use in a coastal city. J. Clean. Prod..

[13] Wang Y., Yang G., Dong Y., Cheng Y., Shang P. (2018). The scale, structure and influencing factors of total carbon emissions from households in 30 provinces of China–Based on the extended STIRPAT model. Energies.

[14] Steg L. (2008). Promoting household energy conservation. Energy Policy.

[15] Zhang X., Wang Y. (2017). How to reduce household carbon emissions: A review of experience and policy design considerations. Energy Policy.

[16] Cason T.N. (2003). Buyer liability and voluntary inspections in international greenhouse gas emissions trading: A laboratory study. Environ. Resour. Econ..

[17] Liu L., Chen C., Zhao Y., Zhao E. (2015). China’s carbon-emissions trading: Overview, challenges and future. Renew. Sustain. Energy Rev..

[18] Lundgren T., Marklund P.O., Samakovlis E., Zhou W. (2015). Carbon prices and incentives for technological development. J. Environ. Manag..

[19] Li W., Jia Z. (2017). Carbon tax, emission trading, or the mixed policy: Which is the most effective strategy for climate change mitigation in China. Mitig. Adapt. Strateg. Glob. Chang..

[20] Fleming D. (1996). Stopping the traffic. Ctry. Life.

[21] Fawcett T., Hvelplund F., Meyer N.I. (2010). Making it personal: Per capita carbon allowances. Generating Electricity in a Carbon-Constrained World.

[22] Chen H. (2014). Development and prospect of personal carbon trading research. China Popul. Resour. Environ..

[23] Fleming D. (1997). Tradable quotas: Using information technology to cap national carbon emissions. Environ. Policy Gov..

[24] Hillman M. (1998). Carbon budget watchers. Town Ctry. Plan..

[25] Fawcett T. (2010). Personal carbon trading: A policy ahead of its time. Energy Policy.

[26] Li J., Wang S., Fan J., Liang L. (2018). An equilibrium model of consumer energy choice using a personal carbon trading scheme based on allowance price. J. Clean. Prod..

[27] Department for Environment, Food and Rural Affairs—DEFRA (2008). Synthesis Report on the Findings from DEFRA’s Pre-Feasibility Study into Personal Carbon Trading.

[28] Eyre N. (2010). Policing carbon: Design and enforcement options for personal carbon trading. Clim. Policy.

[29] Parag Y., Capstick S., Poortinga W. (2011). Policy attribute framing: A comparison between three policy instruments for personal emissions reduction. J. Policy Anal. Manag..

[30] McNamara D., Caulfield B. (2013). Examining the impact of carbon price changes under a personalised carbon trading scheme for transport. Transp. Policy.

[31] Starkey R. (2012). Personal carbon trading: A critical survey part 2: Efficiency and effectiveness. Ecol. Econ..

[32] Taylor P.D., Jonker L.B. (1978). Evolutionarily stable strategies and game dynamics. Math. Biosci..

[33] Weibull J.W. (1995). Evolutionary Game Theory.

[34] Rabanal J.P. (2015). On the evolution of continuous types under replicator and gradient dynamics: Two examples. Dyn. Games Appl..

[35] Zhou J., Nie H., Zhang G., Zhang H. (2011). The evolutionary analysis on enterprise production strategy under the mechanism design theory in low-carbon economy. Sci. Technol. Process Policy.

[36] Friedman D. (1991). Evolutionary games in economics. Econometrica.

[37] Wadud Z. (2011). Personal tradable carbon permits for road transport: Why, why not and who wins. Transp. Res. Part A.

[38] Raux C., Croissant Y., Pons D. (2015). Would personal carbon trading reduce travel emissions more effectively than a carbon tax. Transp. Res. Part D Transp. Environ..

